# Supplementary figures and images for: Identification of patients at risk of new onset heart failure: Utilizing a large statewide health information exchange to train and validate a risk prediction model
Source: PLoS One. 2021 Dec 10;16(12):e0260885. doi: 10.1371/journal.pone.0260885 (PMC8664210; doi:10.1371/journal.pone.0260885)

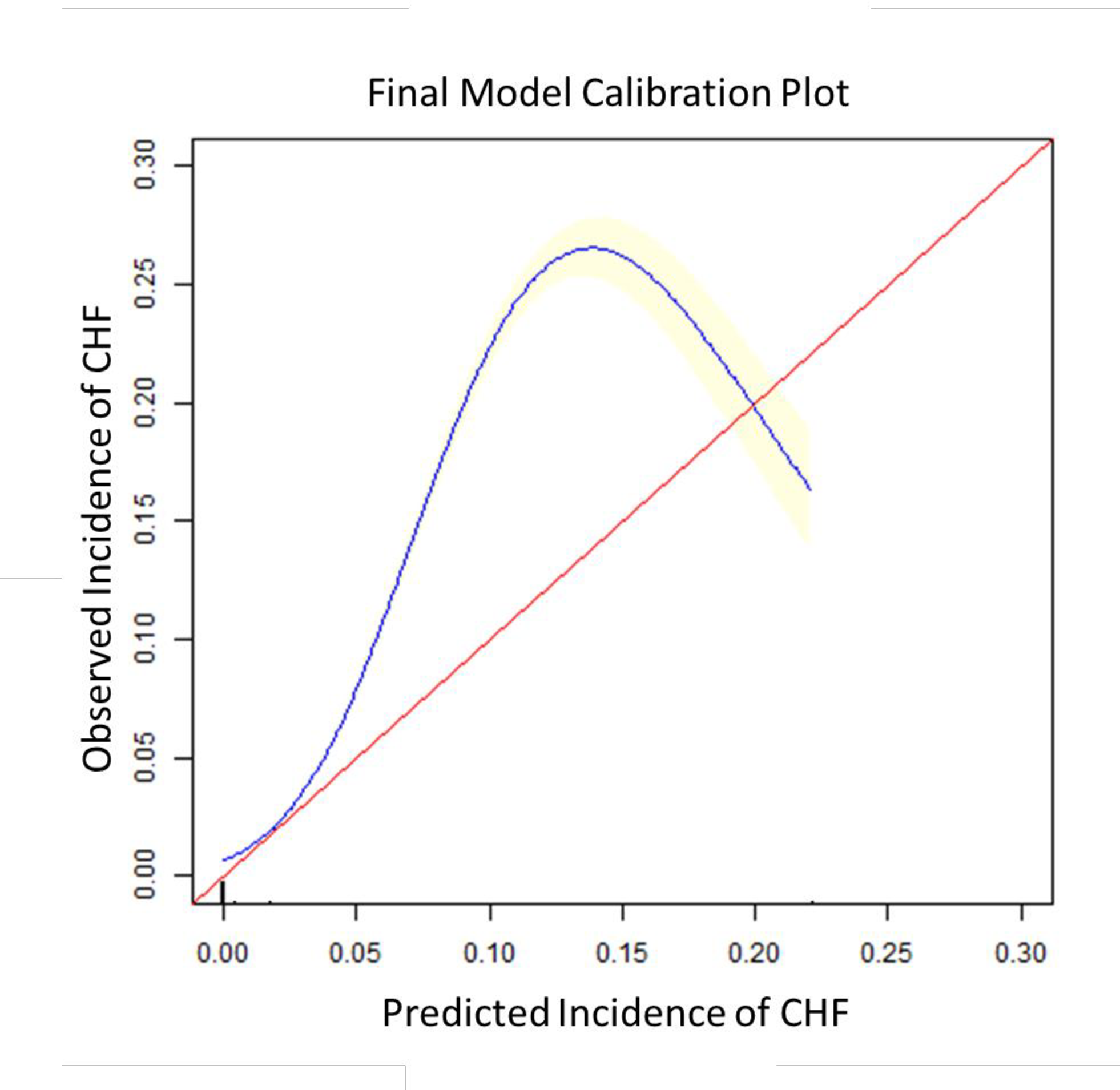

Supplement: S1 Fig — The observed versus expected model predictions across all risk score assignments. Yellow shaded area represents 95% confidence interval. (TIF) [file pone.0260885.s001.tif]

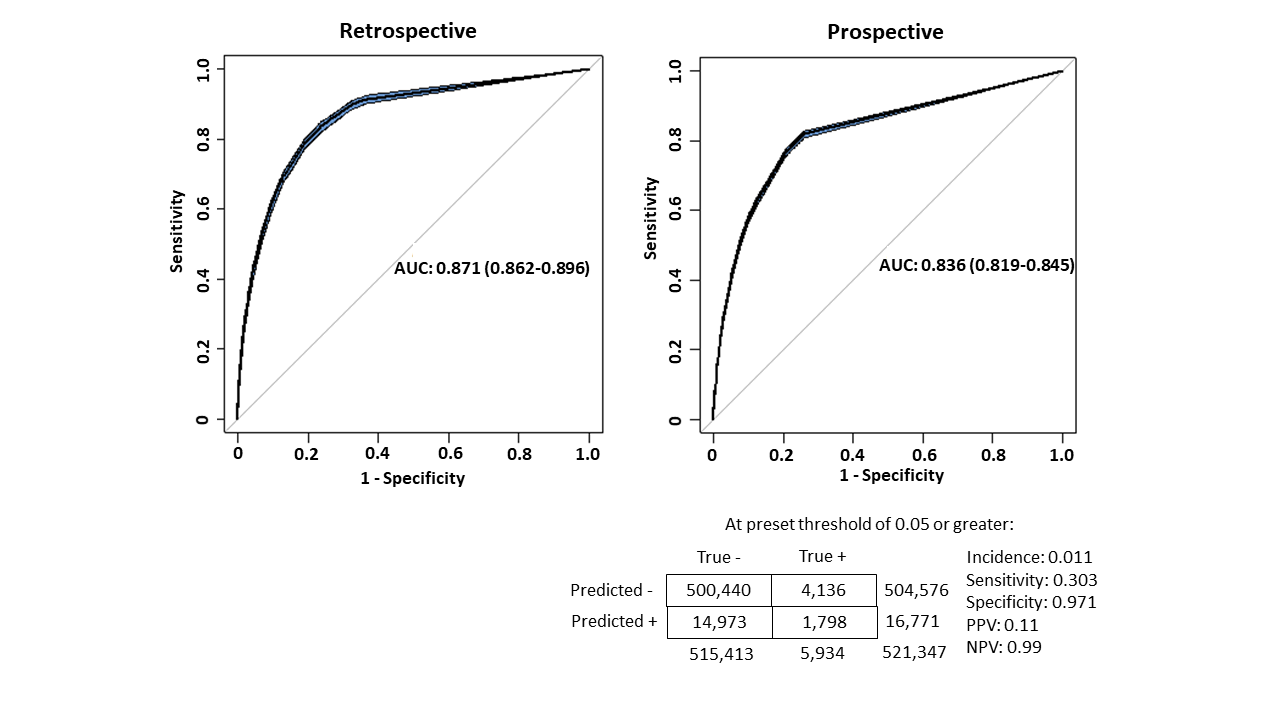

Supplement: S2 Fig — (TIF) [file pone.0260885.s002.tif]

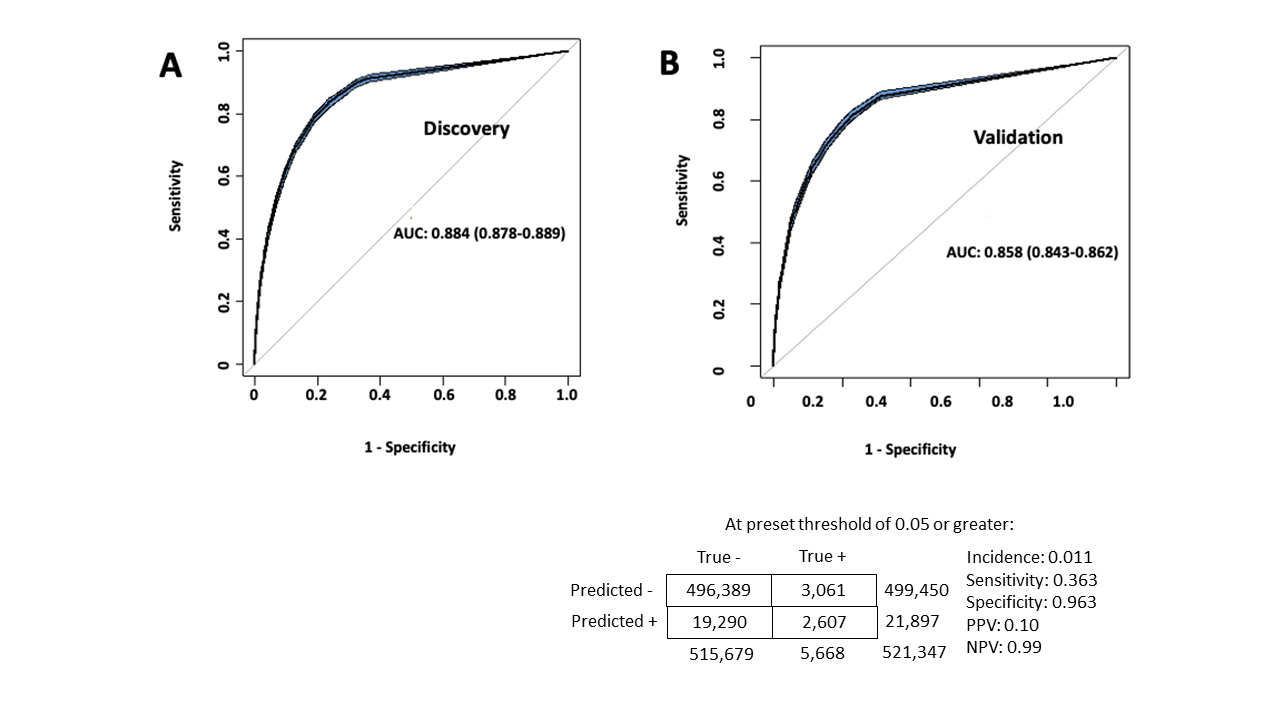

Supplement: S3 Fig — (TIF) [file pone.0260885.s003.tif]
