# Supplementary material for: Identification of patients at risk of new onset heart failure: Utilizing a large statewide health information exchange to train and validate a risk prediction model
Source: PLoS One. 2021 Dec 10;16(12):e0260885. doi: 10.1371/journal.pone.0260885 (PMC8664210; doi:10.1371/journal.pone.0260885)
Supplement: S1 Table — (DOCX) [file pone.0260885.s004.docx]

| Code type | Code |
| --- | --- |
| ICD10-CM | I0981 |
| ICD10-CM | I501 |
| ICD10-CM | I5020 |
| ICD10-CM | I5021 |
| ICD10-CM | I5022 |
| ICD10-CM | I5023 |
| ICD10-CM | I5030 |
| ICD10-CM | I5031 |
| ICD10-CM | I5032 |
| ICD10-CM | I5033 |
| ICD10-CM | I5040 |
| ICD10-CM | I5041 |
| ICD10-CM | I5042 |
| ICD10-CM | I5043 |
| ICD10-CM | I50810 |
| ICD10-CM | I50811 |
| ICD10-CM | I50812 |
| ICD10-CM | I50813 |
| ICD10-CM | I50814 |
| ICD10-CM | I5082 |
| ICD10-CM | I5083 |
| ICD10-CM | I5084 |
| ICD10-CM | I5089 |
| ICD10-CM | I509 |
| ICD9 | 39891 |
| ICD9 | 4280 |
| ICD9 | 4281 |
| ICD9 | 42820 |
| ICD9 | 42821 |
| ICD9 | 42822 |
| ICD9 | 42823 |
| ICD9 | 42830 |
| ICD9 | 42831 |
| ICD9 | 42832 |
| ICD9 | 42833 |
| ICD9 | 42840 |
| ICD9 | 42841 |
| ICD9 | 42842 |
| ICD9 | 42843 |
| ICD9 | 4289 |
