# Supplementary material for: Identification of patients at risk of new onset heart failure: Utilizing a large statewide health information exchange to train and validate a risk prediction model
Source: PLoS One. 2021 Dec 10;16(12):e0260885. doi: 10.1371/journal.pone.0260885 (PMC8664210; doi:10.1371/journal.pone.0260885)
Supplement: S2 Table — (DOCX) [file pone.0260885.s005.docx]

2a

|  | Annual Incidence per 1000 |  |
| --- | --- | --- |
| Age, year | Men | Women |
| 45-54 | 2 | 1 |
| 55-64 | 4 | 2 |
| 65-74 | 9 | 6 |
| 75-84 | 18 | 12 |
| 85-94 | 39 | 31 |

2b

|  | Annual Incidence per 1000 |  |
| --- | --- | --- |
| Age, year | Men | Women |
| 45-54 | 2 | 1 |
| 55-64 | 5 | 3 |
| 65-74 | 11 | 7 |
| 75-84 | 24 | 20 |
| 85-94 | 43 | 34 |
