# Supplementary material for: Identification of patients at risk of new onset heart failure: Utilizing a large statewide health information exchange to train and validate a risk prediction model
Source: PLoS One. 2021 Dec 10;16(12):e0260885. doi: 10.1371/journal.pone.0260885 (PMC8664210; doi:10.1371/journal.pone.0260885)
Supplement: Attachment — Submitted filename: Review.pdf [file pone.0260885.s009.pdf]

Aim:

This paper developed a risk prediction tool to detect incident heart failure in adults using a large state-wide CCHIE from the state of Maine, USA.

A tree-boosting algorithm was trained in order to model the probability of incident heart failure in year two from data collected in year one, and then prospectively validated in year three.

This paper tackles a very important problem that could be solved by using existing routinely collected data. In addition, it shows how difficult could be for an algorithm to predict HF based on administrative and billing codes such ICD10. I enjoyed reading the paper.

The model obtains a high specificity but a very low sensitivity. This could be expected as the classes are highly imbalanced, making this problem a difficult one. If the algorithm classifies everyone as healthy, it will have already very high specificity. I would invite the authors to tackle this problem by using some of the machine learning techniques that deal with imbalanced datasets, for example, using a Class Weighted XGBoost or Cost-Sensitive XGBoost.

In addition, given the vast amount of data (labs, procedures, medicines,...) the authors could use other markers in addition to ICD10 to better define HF. This will include some work with clinicians.

Finally, the authors have seemed to just "plug" the XGBoost to the data without tuning any hyper-parameter. I would also invite the authors to re-visit this and tune some of the most important hyper-parameters of the XGBoost. This could significally improve the performance of the predictive algorithm.

Please find some other suggestions below:

Abstract:

1. Methods and Results: "A tree-boosting algorithm was developed": A tree-boosting algorithm was trained, rather than developed.

2. Conclusions: See my comment below regarding the term "prospectively validated".

Methods

Database and subject selection criteria:

1. I would use the terms "training and test" sets for the sets that you use for training and testing. I am not 100% clear which part of the data you use for training and which one for testing. Explain this in detail with dates and number of records. Table 1 will be ideal for that.

Every machine learning algorithm is tested in some data that the model hasn't seen during the training, but I wouldn't call it "prospectively validated" unless you collect the data prospectively, which as far as I understood, it is not the case here. This paper may be of interest: https://jamanetwork.com/journals/jamanetworkopen/fullarticle/2760438 (Prospective and External Evaluation of a Machine Learning Model to Predict In-Hospital Mortality of Adults at Time of Admission

Nathan Brajer). They used the terms training and test sets to build the model, and then, they prospectively validated by validation the model with real-time data: "The model was integrated into the production electronic health record system and prospectively validated on a cohort of 5273 hospitalizations representing 4525 unique adult patients admitted to hospital A between February 14, 2019, and April 15, 2019." Therefore, in my opinion, you use the data as if it was collected prospectively, which is exactly the idea behind any test set, but you didn't integrate the model into production and validate prospectively.

I would remove the term "prospectively validated" from the manuscript and replace it with "test the algorithm" or validate it in the test set.

2. "Subjects in the model building/training cohort were randomly split into a 2/3 training and 1/3 retrospective prediction group, which was used to train separate models under differing feature sets (see below)." I was confused with the definition of the training and test set. In addition ,Table 1 doesn't specify the dates or the amount of patients (2/3 and 1/3) that falls in each group.

Table 1 says that the training cohort contains 497,470 patients, but Figure 1 says the 497,470 were divided into "observation period" (usually this is the training set) and prediction period (this is NOT usually part of the training set).

Finally, the term "validation cohort" is a bit confusing too, since "validation or development set" is commonly used for hyperparameter tuning. As stated above, I would change it to training and test. If validation cohort is preferred, please state clearly, with dates and number of patients, what sets you used for training, test and external testing or "validation". But I don't think it is either external (since the data comes from the same system) or prospective (as it is not collected prospectively).

Feature selection and preprocessing:

1. Did you convert the ICD9 to ICD10 codes? You talked about using 5 or 3 digits with the ICD10. How do you deal with the ICD9 codes?

2. "Finally, we performed a univariate filtering step to eliminate features associated

78 with the outcome with a chi-squared test p-value >0.2." You could use XGBoost for both purposes, 1) feature/dimensionality reduction (https://machinelearningmastery.com/feature-importance-and-feature-selection-with-xgboost-in-python/) and 2) Final model. It would be interesting to see which features the XGBoost presents as the most relevant.

3. Did not you include patient characteristics such as gender? I think males tend to have higher incidence of HF.

4. "Medications were mapped to medication class using the Established Pharmacologic Class coding system". Were medications treated as binary 1-Medication was taken, 0 -Wasn't used, or did you use the amount of units, tablets,...?

In supplementary Table 3, the medications contain this text: Patient had *** medications , which makes me think you consider a continuous number. Please clarify.

5. Exactly the same for lab test, for example "Patient had *** abnormal laboratory tests (INR in Blood by Coagulation assay) in the last 12 months". Maybe a binary flag will be enough.

6. In addition, I think it would help to present this supplementary table 3 by groups: medicines, laboratory test, demographics, ICD10 Umbrella, CPT4 Code. It would be very interesting to see what features are relevant for the algorithm.

Outcome definition:

1. "Development of HF was defined as new assignment of an ICD-10 code for HF". Therefore, no ICD9 codes were considered for the definition of HF. am I right? I assumed all the systems were using ICD10 then. Supplementary table 1 includes some ICD9 codes, which was confusing.

2. As stated above, please clarify the training and test sets.

Model construction:

1. Hyper-parameter tuning: I haven't seen any reference to the hyper-parameters of the model. As per the API, there are many hyper-parameters that can be tuned: https://xgboost.readthedocs.io/en/latest/parameter.html. That is, a "development" set (in addition to the training and test set) should be put aside to tune these hyper-parameters. Alternatively, cross validation could be used in the training set using techniques such as grid search or random search to tune those hyperparameters.

a. Why did you decide to change the max_depth from the default value (6) to 5?

b. Why didn't you tune any of the others hyper-parameters or a subset of them? This could change the performance by much. For example, learning rate, number of estimators, type of regularization (L1 or L2), ...

2. Reproduction of the results: Which library, version and software did you use? Which type of machine?

Results:

1. In the methods you wrote: "Finally, we performed a univariate filtering step to eliminate features associated

78 with the outcome with a chi-squared test p-value >0.2." but in the results, it seems that the features were chosen by the XGBoost algorithm: "Of the 43,906 possible data features before feature reduction techniques were performed, the boost algorithm selected 339 for inclusion in the final model (Supplemental Table 3)." Please clarify.

2. I didn't understand this sentence: "The model also selected as weak classifiers features such as undergoing eye

surgery, laxative use, abnormal iron levels, and vitamin D use, to name a few."

Figure 2:

The confusion matrix was confusing. Please use standard names: Predicted versus True/
