# Supplementary material for: Identification of patients at risk of new onset heart failure: Utilizing a large statewide health information exchange to train and validate a risk prediction model
Source: PLoS One. 2021 Dec 10;16(12):e0260885. doi: 10.1371/journal.pone.0260885 (PMC8664210; doi:10.1371/journal.pone.0260885)
Supplement: Attachment — Submitted filename: Response reviewers 2.docx [file pone.0260885.s008.docx]

Dear Richard Ibañez Dilla,

Please find our response to the items below in **bold.**

Sincerely,

Son Duong

PONE-D-21-10920R1
A prospectively validated novel risk prediction model for new onset heart failure utilizing a large statewide health information exchange
Dr. Son Duong

Dear Dr. Duong,

We've checked your submission and before we can proceed, we need you to address the following issues:

1. Please review your reference list to ensure that it is complete and correct. If you have cited papers that have been retracted, please include the rationale for doing so in the manuscript text, or remove these references and replace them with relevant current references. Any changes to the reference list should be mentioned in the rebuttal letter that accompanies your revised manuscript. If you need to cite a retracted article, indicate the article’s retracted status in the References list and also include a citation and full reference for the retraction notice.

**We have reviewed the reference list and determined that it is complete, correct, and no retracted articles are included.**

2. Thanks for providing the requested data sharing information. Based on these details, we’ve drafted for your approval the following proposed Data Availability Statement:

"The work was performed under a business arrangement between HealthInfoNet (http://www.hinfonet.org), the operators of the Maine Health Information Exchange and HBI Solutions, Inc. (HBI) located in California. HIN is a steward of the data on behalf of its members which includes health systems, hospitals, medical groups and federally qualified health centers. The data is owned by the HIN members, not HIN. HIN is responsible for security and access to its members' data and has established data service agreements (DSAs) restricting unnecessary exposure of information. HIN and its board (comprised from a cross section of its members) authorized the use of the de-identified data for this research, as the published research helps promote the value of the HIE and value to Maine residents.

The research was conducted on HIN technology infrastructure, and the researchers accessed the de-identified data via secure remote methods. All data analysis and modeling for this manuscript was performed on HIN servers and data was accessed via secure connections controlled by HIN.

Access to the data used in the study requires secure connection to HIN servers and should be requested directly to HIN. Researchers may contact Shaun T. Alfreds at salfreds@hinfonet.org to request data. Data will be available upon request to all interested researchers. HIN agrees to provide access to the de-identified data on a per request basis to interested researchers. Future researchers will access the data through exact the same process as the authors of the manuscript."

Please confirm whether this information is accurate, and whether we may update your Data Availability Statement on your behalf.

**We confirm that this information is accurate and you may update the Data Availability Statement on our behalf.**

We've returned your manuscript to your account. Please resolve these issues and resubmit your manuscript within 21 days. If you need more time, please email the journal office at plosone@plos.org. We are happy to grant extensions of up to one month past this due date. If we do not hear from you within 21 days, we will withdraw your manuscript.

Please log on to PLOS Editorial Manager at https://www.editorialmanager.com/pone/ to access your manuscript. You will find your manuscript in the 'Submissions Sent Back to Author' link under the New Submissions menu. Be sure to remove your previous manuscript file if you are uploading a new file in response to these requests. After you've made the changes requested above, please be sure to view and approve the revised PDF after rebuilding the PDF to complete the resubmission process.

We are requesting these changes to comply with the PLOS ONE submission guidelines (https://journals.plos.org/plosone/s/submission-guidelines). Please note that we won't send your manuscript for review until you have resolved the above requests.

Thank you for submitting your work to PLOS ONE and supporting our mission of Open Science.


Kind regards,

Richard Ibañez Dilla
PLOS ONE

__________________________________________________
In compliance with data protection regulations, you may request that we remove your personal registration details at any time. (Use the following URL: https://www.editorialmanager.com/pone/login.asp?a=r). Please contact the publication office if you have any questions.
