# Supplementary material for: Identification of patients at risk of new onset heart failure: Utilizing a large statewide health information exchange to train and validate a risk prediction model
Source: PLoS One. 2021 Dec 10;16(12):e0260885. doi: 10.1371/journal.pone.0260885 (PMC8664210; doi:10.1371/journal.pone.0260885)
Supplement: Attachment — Submitted filename: 9 2021 PONE rebuttal letter v2.docx [file pone.0260885.s010.docx]

**PONE-D-21-10920R1 REBUTTAL LETTER**

Title “A prospectively validated novel risk prediction model for new onset heart failure utilizing a large statewide health information exchange”

**RESPONSE:** Title has been revised as “Case finding for patients at risk of new onset heart failure: utilizing a large statewide health information exchange EHR data to train and validate a risk prediction model”.
===================================

**RESPONSE: we** logged on to <https://www.editorialmanager.com/pone/> and select the 'Submissions Needing Revision' folder to include the following items when submitting our revised manuscript:

- A rebuttal letter that responds to each point raised by the academic editor and reviewer(s). This letter was uploaded as a separate file labeled 'Response to Reviewers'.
- A marked-up copy of your manuscript that highlights changes made to the original version. A separate file labeled 'Revised Manuscript with Track Changes' was uploaded.
- An unmarked version of your revised paper without tracked changes. A separate file labeled 'Manuscript' without track was uploaded.

**RESPONSE to REVIEWER 2:**

- The manuscript itself lacks a lot of literature review on related works.

**RESPONSE:** Additional literature of related works has been added to the METHODS per suggestion

- The authors should compare the performance results to previous studies on the same dataset.

**RESPONSE:** Our HIE dataset has not been analyzed by other groups yet, therefore, it would be technically challenging to compare on the same HIE dataset.

- The authors should propose more feature selection techniques to find out the optimal ones.

How did the authors perform hyperparameter optimization of the models?

**RESPONSE:** A new method section “Model revisited” was added to address these points.

- Machine learning-based model (i.e., XGBoost) has been used in previously biomedical studies such as PMID: 31987913 and PMID: 32942564. Thus, the authors are suggested to refer to more works in this description to attract a broader readership.

RESPONSE: The method section was revised to reference PMID: 31987913 and PMID: 32942564.

- There must have space before reference number.

**RESPONSE:** The text was revised to remove space before the reference number.

**RESPONSE to REVIEWER 3:**

I would invite the authors to tackle this problem by using some of the machine learning techniques that deal with imbalanced datasets, for example, using a Class Weighted XGBoost or Cost-Sensitive XGBoost.

Finally, the authors have seemed to just "plug" the XGBoost to the data without tuning any hyper-parameter. I would also invite the authors to re-visit this and tune some of the most important hyper-parameters of the XGBoost. This could significally improve the performance of the predictive algorithm.

**RESPONSE:** A new section under “Exploratory Model Analyses” was added to address these points.

Please find some other suggestions below:

Abstract:
1. Methods and Results: "A tree-boosting algorithm was developed": A tree-boosting algorithm was trained, rather than developed.

**RESPONSE:** revision was made throughout the main text.

Methods

Database and subject selection criteria:

1. I would use the terms "training and test" sets for the sets that you use for training and testing. I am not 100% clear which part of the data you use for training and which one for testing. Explain this in detail with dates and number of records. Table 1 will be ideal for that.

Every machine learning algorithm is tested in some data that the model hasn't seen during the training, but I wouldn't call it "prospectively validated" unless you collect the data prospectively, which as far as I understood, it is not the case here. This paper may be of interest: https://jamanetwork.com/journals/jamanetworkopen/fullarticle/2760438 (Prospective and External Evaluation of a Machine Learning Model to Predict In-Hospital Mortality of Adults at Time of Admission Nathan Brajer). They used the terms training and test sets to build the model, and then, they prospectively validated by validation the model with real-time data: "The model was integrated into the production electronic health record system and prospectively validated on a cohort of 5273 hospitalizations representing 4525 unique adult patients admitted to hospital A between February 14, 2019, and April 15, 2019." Therefore, in my opinion, you use the data as if it was collected prospectively, which is exactly the idea behind any test set, but you didn't integrate the model into production and validate prospectively.

I would remove the term "prospectively validated" from the manuscript and replace it with "test the algorithm" or validate it in the test set.

2. "Subjects in the model building/training cohort were randomly split into a 2/3 training and 1/3 retrospective prediction group, which was used to train separate models under differing feature sets (see below)." I was confused with the definition of the training and test set. In addition ,Table 1 doesn't specify the dates or the amount of patients (2/3 and 1/3) that falls in each group.

Table 1 says that the training cohort contains 497,470 patients, but Figure 1 says the 497,470 were divided into "observation period" (usually this is the training set) and prediction period (this is NOT usually part of the training set).

Finally, the term "validation cohort" is a bit confusing too, since "validation or development set" is commonly used for hyperparameter tuning. As stated above, I would change it to training and test. If validation cohort is preferred, please state clearly, with dates and number of patients, what sets you used for training, test and external testing or "validation". But I don't think it is either external (since the data comes from the same system) or prospective (as it is not collected prospectively).

I would remove the term "prospectively validated" from the manuscript and replace it with "test the algorithm" or validate it in the test set

2. "Subjects in the model building/training cohort were randomly split into a 2/3 training and 1/3 retrospective prediction group, which was used to train separate models under differing feature sets (see below)." I was confused with the definition of the training and test set. In addition ,Table 1 doesn't specify the dates or the amount of patients (2/3 and 1/3) that falls in each group.

**RESPONSE:**

All the above points are related to the clarification of discovery and validation analytics. Therefore, we address collectively here. We summarize the reviewer’s input into two perspectives: (1) confusion as to the meaning of “prospective”; (2) need additional clarification for the retrospective (training) and prospective (validation) cohorts (Table 1).

We agree with the reviewer that the manuscript needs additional clarification—overall we meant prospective to mean the algorithim was validated on data in a subsequent time period, but acknowledge that this is an unclear and potentially inaccurate definition. We therefore have removed all references to prospective validation and instead simply refer to this process as “validation”.

1. Title “A prospectively validated novel risk prediction model for new onset heart failure utilizing a large statewide health information exchange” was revised as “Identification of patients at risk of new onset heart failure: utilizing a large statewide health information exchange to train and validate a risk prediction model”.
2. In the method section, we used the terms of “discovery cohort” (i.e the old retrospective) and “validation cohort” (i.e the old prospective) to clarify. The discovery cohort was randomly partitioned into 1/3 for training and cross validation, 1/3 for calibration, and 1/3 for blind testing for performance evaluation. The final model performance was then measured on the subsequent year of data: the “validation” cohort.
3. We added an “Exploratory analysis” section to the methods and results. In these analyses we incorporated several reviewer suggestison on model development and tuning and reported the improved results.
4. Table 1 was updated in the main text to include more subject information.

1. Did you convert the ICD9 to ICD10 codes? You talked about using 5 or 3 digits with the ICD10. How do you deal with the ICD9 codes?

**RESPONSE:** Method section was revised to clarify that we only used ICD9 codes to exclude patients with historical record of having heart failure.

2. "Finally, we performed a univariate filtering step to eliminate features associated
78 with the outcome with a chi-squared test p-value >0.2." You could use XGBoost for both purposes, 1) feature/dimensionality reduction (<https://machinelearningmastery.com/feature-importance-and-feature-selection-with-xgboost-in-python/>) and 2) Final model. It would be interesting to see which features the XGBoost presents as the most relevant.

**RESPONSE:** We incorporated this suggestion and reported this under additional exploratory analyses. The method text was revised to add a new section and the results were summarized in the limitation section and the supplementary materials. A table in the supplemental file (S3) is provided with all features selected by their data type and relative importance rank as requested.

3. Did not you include patient characteristics such as gender? I think males tend to have higher incidence of HF.

**RESPONSE:** Gender is one of the discriminative features (Table 2, importance rank 17) used by XGboost in the trained model.

4. "Medications were mapped to medication class using the Established Pharmacologic Class coding system". Were medications treated as binary 1-Medication was taken, 0 -Wasn't used, or did you use the amount of units, tablets,...?
In supplementary Table 3, the medications contain this text: Patient had *** medications , which makes me think you consider a continuous number. Please clarify.

**RESPONSE:** Medications were treated as features with binary values. Supplementary Table 3 was revised for clarity.

5. Exactly the same for lab test, for example "Patient had *** abnormal laboratory tests (INR in Blood by Coagulation assay) in the last 12 months". Maybe a binary flag will be enough.

**RESPONSE:** Laboratory tests were treated as features with binary values. Supplementary Table 3 was revised for clarity.

6. In addition, I think it would help to present this supplementary table 3 by groups: medicines, laboratory test, demographics, ICD10 Umbrella, CPT4 Code. It would be very interesting to see what features are relevant for the algorithm.

**RESPONSE:** Supplementary table 3 was revised as suggested.

Outcome definition:
1. "Development of HF was defined as new assignment of an ICD-10 code for HF". Therefore, no ICD9 codes were considered for the definition of HF. am I right? I assumed all the systems were using ICD10 then. Supplementary table 1 includes some ICD9 codes, which was confusing.

**RESPONSE:** Only ICD-10 was used in the modeling process. However, when enrolling the patients, we excluded patients with historical HF events encoded by ICD-9. We used ICD-9 codes under this context.

2. As stated above, please clarify the training and test sets.

Model construction:
1. Hyper-parameter tuning: I haven't seen any reference to the hyper-parameters of the model. As per the API, there are many hyper-parameters that can be tuned: <https://xgboost.readthedocs.io/en/latest/parameter.html>. That is, a "development" set (in addition to the training and test set) should be put aside to tune these hyper-parameters. Alternatively, cross validation could be used in the training set using techniques such as grid search or random search to tune those hyperparameters.
a. Why did you decide to change the max_depth from the default value (6) to 5?
b. Why didn't you tune any of the others hyper-parameters or a subset of them? This could change the performance by much. For example, learning rate, number of estimators, type of regularization (L1 or L2), ...

**RESPONSE:** We have expanded the methods section to include the hyper-parameter tuning process using a grid search method and the parameters chosen under “model construction and Tuning”

2. Reproduction of the results: Which library, version and software did you use? Which type of machine?

**RESPONSE:** Method section was revised to include a new sub section to address these points.

Results:
1. In the methods you wrote: "Finally, we performed a univariate filtering step to eliminate features associated 78 with the outcome with a chi-squared test p-value >0.2." but in the results, it seems that the features were chosen by the XGBoost algorithm: "Of the 43,906 possible data features before feature reduction techniques were performed, the boost algorithm selected 339 for inclusion in the final model (Supplemental Table 3)." Please clarify.

**RESPONSE:** The method section was revised to clarify that the first step was to remove features with a univariate chi-squared test, and the second step was to allow the XGBoost algorithim to select important features with an importance gain greater than 0.

2. I didn't understand this sentence: "The model also selected as weak classifiers features such as undergoing eye
surgery, laxative use, abnormal iron levels, and vitamin D use, to name a few."
**RESPONSE:** This sentence was removed.

Figure 2:
The confusion matrix was confusing. Please use standard names: Predicted versus True

**RESPONSE:** The figure was revised as suggested as suggested.

Reviewer #4: The authors report the development of risk prediction tool for development of HF in adults using a large state-wide CCHIE from the state of Maine. In terms of data, the author collects enough data for modeling and analysis; in terms of algorithm, the author uses the classical machine learning algorithm xgboost to model the probability of incident-HF in year two from data collected in year one, and then prospectively validated in year three. Here are some questions that may need to be explained:
1.Throughout the data set, the positive data (disease +) is much less than the negative data (disease -), only about 1%, the data is highly uneven. For this kind of binary classification problem of data imbalance, we should first down-sample the (disease -) data to keep the two types of data as balanced as possible before modeling. Because of the data imbalance, the Sensitivity and PPV shown in the confusion matrix of Figure2 are not high enough. How does the author think about this problem?

**RESPONSE:**  The goal of our study was to create a clinically applicable model using real world EMR data to model the future clinical outcomes. We agree that it is common in data mining to have an imbalanced dataset. However, in our study we felt that the PPV and sensitivity were actually very good for a real-world test meant to identify potential cases of heart failure, not to definitively diagnose heart failure. The goal here is NOT for binary classification but to find patients at risk for targeted clinical outcomes. However, we agree that class imbalances do need to be accommodated in our models and therefore we included additional analysis to do so under “Exploratory analyses”. The results were summarized in the supplementary section. Indeed, we observed the modest improvement (*p value,* 0.01) of the overall predictive performance (ROC AUC), though we note that the PPV was unchanged and the specificity of the test was actually decreased. We elaborate in the limitations section that the choice of optimal test ultimately lies in the clinical use of the test, and that continued tuning/optimization of a prediction model to meet the need in question is required for implementation.

2.On page 6 start from Line 89, The authors use XGboost algorithm to build two models, but the reason for choosing this algorithm needs to be supplemented, why there is no other algorithm, such as SVM or fully connected neural network and so on.

**RESPONSE:** We have tried different modeling algorithms (data not shown) including SVM and ultimately chose XGBoost for its modeling performance and computing efficiency.

3.Among the excluded patients, only the diagnosis or data of the previous year were excluded. If the patients did not come to see a doctor because of HF in the previous year, but had a previous history of HF (such as diagnosed earlier), how to exclude them? Will them be mistakenly included?

**RESPONSE:** In our study we excluded all patients with any prior history of HF in their record (even before one-year). However if a patient were new to the system and carried a diagnosis of HF from another system, than there is a potential for including them as at-risk for developing HF when in fact they already had that diagnosis. We have added this clarification to the limitation section.

4.The table 1 baseline data are too simple, so we should compare the baseline data between the training group and the validation group, and whether there is any difference between the heart failure group and the non-heart failure group combined with the most important feature found by table 2.

**RESPONSE:** Table 1 was revised as suggested.

Reviewer #5: The manuscript describes an original application of machine learning for new-onset heart failure prediction in a 1-year timeframe on a large cohort of subjects.

Based on the manuscript in its current form, I do not have sufficient elements to know whether statistical analysis (especially machine learning) has been performed appropriately. The level of detail in which the methods and experimental procedures are described should be increased.

Most importantly, please add more details related to the machine learning experiments. For example:
- Is data reduction applied before the classification? If so, was it done only on the training set to avoid information leakage?

**RESPONSE:**

We applied feature engineering pipeline to preprocess steps that transform raw data into features that can be used in machine learning algorithms on both training set and test set, and also perform feature selection based on transformed features in order to eliminate feature with low variation only for training set. And there should be no information leakage since no subject level data reduction was applied. For feature engineering, since raw data collection resulted in a massive number of potential coding features. Domain knowledge mapping was applied, medications were mapped to medication class using the Established Pharmacologic Class coding system; laboratory data were aggregated into “abnormal” and “normal” binary categories; and we performed an experiment to determine whether aggregating the 5-digit ICD-CM10-CM codes into the 3-digit code to the left of the decimal (henceforth known as the ICD-10 subheader code) would improve model performance and reduce dimensionality. Finally, we performed a univariate filtering step to eliminate features associated with the outcome with a chi-squared test p-value >0.2.

We revised the method section to add a subsection under “Exploratory Analysis” to followed this and other reviewers’ input to fine tune the model and results were described in the Potential limitation section to compare the improvement and effectiveness.

- What kind of cross-validation was used for model development?

**RESPONSE:** 10-fold cross-validation was used when we used 1/3 of the discovery cohort to train the model. Relevant method section was revised.

- How was the final model derived?

**RESPONSE:** Method section was revised to clarify: “Subjects in the discovery cohort were randomly split into 1/3 training, 1/3 calibration, and 1/3 performance testing groups. The modeling, calibration and performance blind testing processes were used to minimize over-optimism of the test performance characteristics.”

- How were the XGBoost parameters chosen?

**RESPONSE:** We have clarified this in the methods section under “Model construction and tuning”: After hyper-parameter fine tuning process, the optimized hyper-parameter combination was found based on best model performance (the learning rate was set to 0.3, the depth of each tree was set to 5 and number of estimators was set to 500). Then, these optimized parameters were applied for XGBoost algorithm on entire training set to derive final model.

- The data set classes appear to be highly imbalanced: was this taken into account in model development?

**RESPONSE:** This was raised by another reviewer as well. We addressed this in exploratory analyses.

Please provide further details about the "data reduction techniques to reduce dimensionality" mentioned in the Methods section. The initial number of features is reported only in the Results section: I suggest mentioning it also in the Methods section, "Feature selection and preprocessing", together with the number of features resulting after dimensionality reduction and the univariate filtering step.

**RESPONSE:** The method section was revised to clarify as suggested in the “Feature standardization, reduction, and selection”.

=============================================

Please specify what criterion was followed to aggregate lab data into abnormal/normal.

**RESPONSE:** Method section was revised to clarify this. “Laboratory data were provided from the HIE as “abnormal” and “normal” binary categories due data interoperability challenges requiring raw test values to be converted to binary abnormal/normal categorical variables via comparing test result value against the corresponding care providers’ test normal reference range.”

When the AUC values are first reported, e.g. "0.797 [0.790-0.803]" etc, the "95% CI" statement inside the brackets is missing. Moreover, are the results reported as mean or median plus CI?

**RESPONSE:** The text was revised as suggested. The results reported as median with 95% CI.

Minors:
- Please check the manuscript for proper spacing between words and references, spelling ("others models", "this algorithm could integrate into the HIE", etc.), and punctuation.

**RESPONSE:** The texts were revised accordingly.

7. PLOS authors have the option to publish the peer review history of their article ([what does this mean?](https://journals.plos.org/plosone/s/editorial-and-peer-review-process#loc-peer-review-history)). If published, this will include your full peer review and any attached files.


If you choose “no”, your identity will remain anonymous but your review may still be made public.


**Do you want your identity to be public for this peer review?** For information about this choice, including consent withdrawal, please see our [Privacy Policy](https://www.plos.org/privacy-policy).

Reviewer #2: No

Reviewer #3: **Yes:** Oscar Perez-Concha

Reviewer #4: No

Reviewer #5: No

[NOTE: If reviewer comments were submitted as an attachment file, they will be attached to this email and accessible via the submission site. Please log into your account, locate the manuscript record, and check for the action link "View Attachments". If this link does not appear, there are no attachment files.]

While revising your submission, please upload your figure files to the Preflight Analysis and Conversion Engine (PACE) digital diagnostic tool, <https://pacev2.apexcovantage.com/>. PACE helps ensure that figures meet PLOS requirements. To use PACE, you must first register as a user. Registration is free. Then, login and navigate to the UPLOAD tab, where you will find detailed instructions on how to use the tool. If you encounter any issues or have any questions when using PACE, please email PLOS at [figures@plos.org](mailto:figures@plos.org). Please note that Supporting Information files do not need this step.
